# Hyperostosis frontalis interna presenting as a forehead scar in a young male

**DOI:** 10.1002/ccr3.7256

**Published:** 2023-04-23

**Authors:** Ahtesham Khizar, Waleed Shahzad, Pradhumna Kumar Yadav

**Affiliations:** ^1^ Department of Neurosurgery Pakistan Institute of Medical Sciences Islamabad Pakistan; ^2^ Department of Neurosurgery Janaki Health Care and Research Center Janakpur Nepal

**Keywords:** forehead scar, hyperostosis frontalis interna

## Abstract

Hyperostosis frontalis interna is a benign overgrowth of the inner table of the frontal bone. Exact etiology is unknown. The condition is often an incidental finding and requires no treatment unless there are neurological signs and symptoms.

A 25‐year‐old man came to our outpatient clinic with a 3‐year‐old forehead scar (Figure 1A,B) There was no history of trauma, and no other signs or symptoms to go along with it. He had a non‐contrast computed tomography brain scan, which revealed thickening of the inner frontal bone, indicating hyperostosis frontalis interna (Figure. 2).

**FIGURE. 1:**
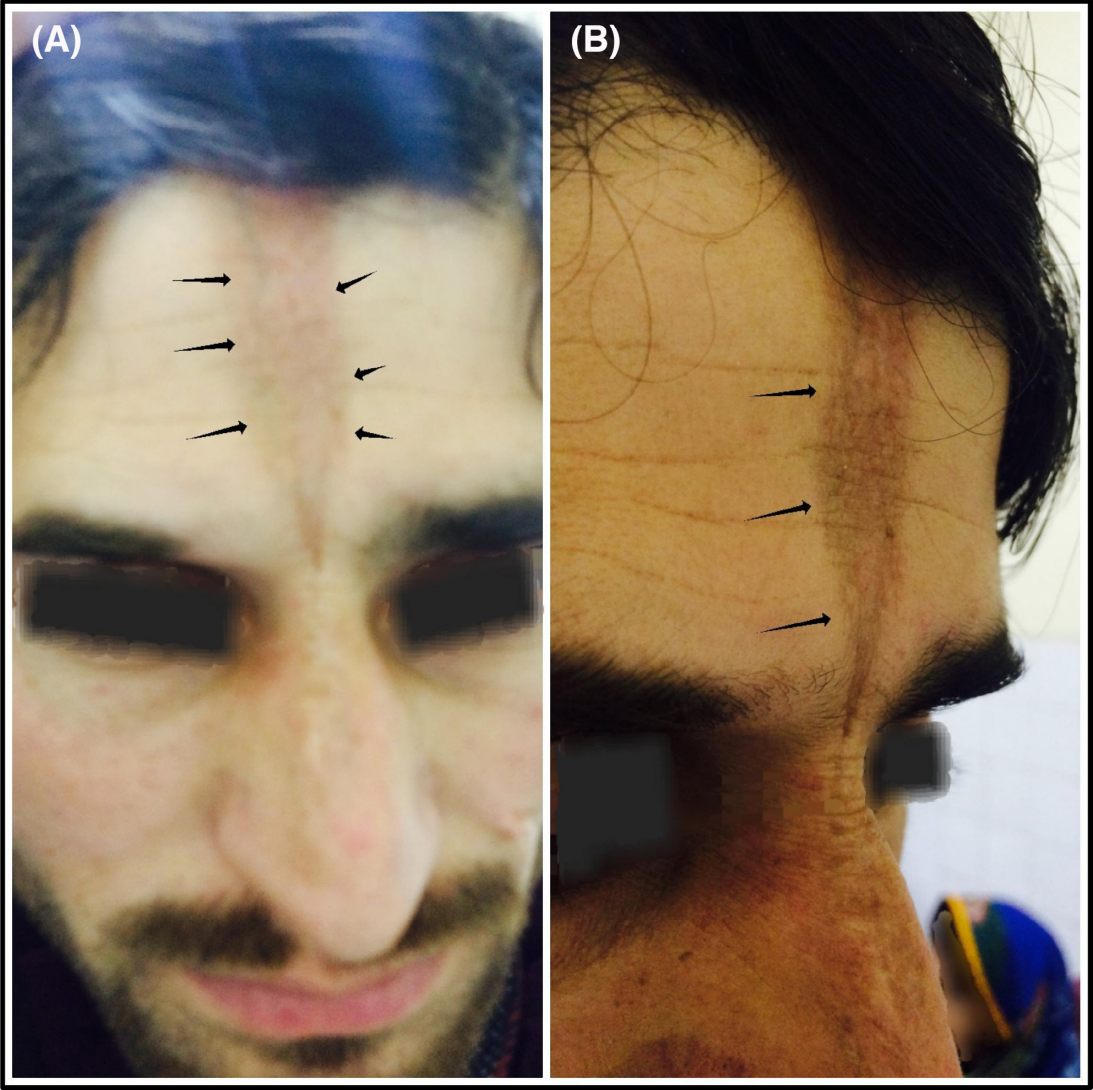
(A,B) Forehead scar in a young male with Hyperostosis Frontalis Interna.

**FIGURE. 2:**
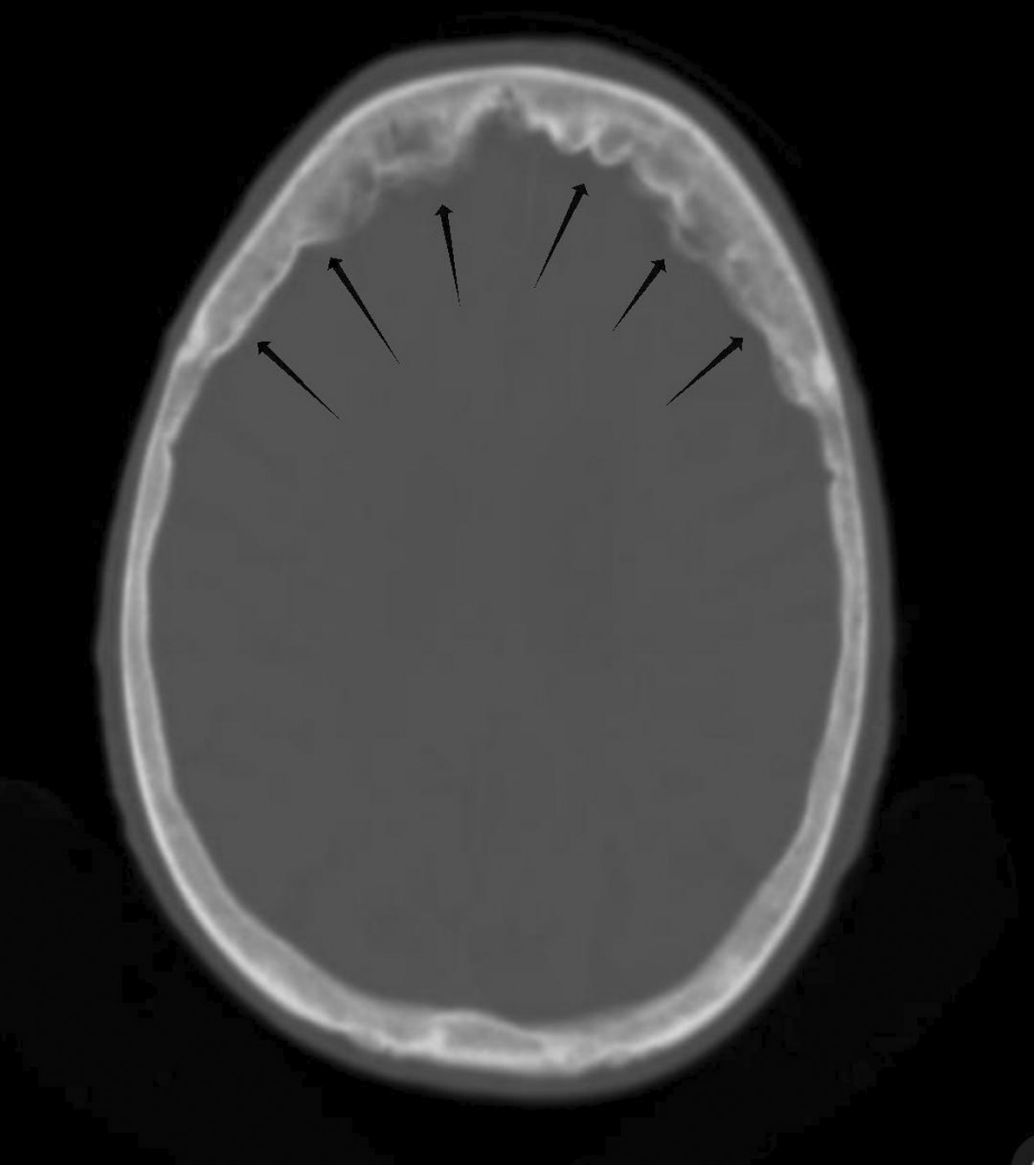
Non‐contrast axial computed tomography (CT) brain showing hyperostosis of inner table of frontal bone.

Hyperostosis frontalis interna, first described in 1765 by Santorini and Morgagni, is characterized by benign overgrowth of the inner table of the frontal bone.^1^ The exact etiology is unknown. The condition is generally of no clinical significance and is often an incidental finding. The growth of the bone is not malignant. Often, the patient is completely unaware of the problem throughout his or her life. This overgrowth of bone is much more common in women than in men. It also appears to be more common in elderly women approaching menopause. Compression by calvarial thickening may lead to cerebral atrophy and may present with cognitive impairment, neuropsychiatric symptoms, headaches, and epilepsy. It is usually symmetrical and bilateral, and it can spread to the parietal bones. Sessile or nodular thickening of the skull can impact the bone in a focused or diffuse manner.^2^

## AUTHOR CONTRIBUTIONS

**Ahtesham Khizar:** Conceptualization; data curation; methodology; project administration; visualization; writing – original draft; writing – review and editing. **Waleed Shahzad:** Formal analysis; investigation; resources; software. **Pradhumna Kumar Yadav:** Formal analysis; investigation; project administration; supervision; validation.

## CONFLICT OF INTEREST STATEMENT

The authors declare no conflict of interest.

## ETHICS STATEMENT

Not applicable.

## CONSENT

Written informed consent was obtained from the patient for publication of images.

## Data Availability

All the data is available within the article.
